# Multilocus microsatellite signature and identification of specific molecular markers for *Leishmania aethiopica*

**DOI:** 10.1186/1756-3305-6-160

**Published:** 2013-06-04

**Authors:** Nigatu Kebede, Steve Oghumu, Alemayehu Worku, Asrat Hailu, Sanjay Varikuti, Abhay R Satoskar

**Affiliations:** 1 Aklilu Lemma Institute of Pathobiology, Addis Ababa University, Addis Ababa, Ethiopia; 2 Department of Pathology, College of Medicine, Ohio State University Wexner Medical Center, Columbus OH 43210, USA; 3 Department of Epidemiology and Biostatistics, School of Public Health, Addis Ababa University, Addis Ababa, Ethiopia; 4 Department of Oral Biology, College of Dentistry, Ohio State University, Columbus, OH 43210, USA; 5 Department of Microbiology, Immunology and Parasitology, School of Medicine, Addis Ababa University, Addis Ababa, Ethiopia

**Keywords:** Leishmania aethiopica, Microsatellite markers, Ethiopia

## Abstract

**Background:**

Leishmaniasis is a clinically and epidemiologically diverse zoonotic disease caused by obligatory, intracellular protozoan parasites of the genus *Leishmania*. Cutaneous leishmaniasis is the most widely distributed form of the disease characterized by skin lesions. *Leishmania aethiopica* is considered the predominant etiological agent in Ethiopia. The current study was aimed at developing multilocus microsatellite markers for *L*. *aethiopica* isolated from human cutaneous leishmaniasis patients in Ethiopia.

**Results:**

*L*. *aethiopica* parasites for the study were obtained from Ethiopia and laboratory analysis was conducted at The Ohio State University. DNA was extracted from cultured parasites and an internal transcribed spacer located at the ribosomal region of *L*. *aethiopica* genomic DNA was PCR amplified for species identification. Microsatellite markers were identified using multilocus microsatellite typing. We generated an enriched genomic library, and using Primer3 software, designed PCR primers to amplify sequences flanking the detected microsatellites. Subsequent screening of the amplified markers for length variations was performed by gel electrophoresis.

Using a variety of molecular methods, 22 different microsatellite markers were identified and tested for typing *L*. *aethiopica* strains using a number of clinical isolates. Of the 22 markers tested, 5 were polymorphic and showed distinctive multilocus genotypes, classifying them into four clusters. One marker was found to be specific for *L*. *aethiopica*, discriminating it from other species of *Leishmania*.

**Conclusion:**

Multilocus microsatellite typing using the markers developed in this study could be useful for epidemiological and population genetic studies of strains of *L*. *aethiopica* in order to investigate the structure and dynamics of the corresponding natural foci. It could also help to answer specific clinical questions, such as the occurrence of local and diffuse lesions, strain correlates of parasite persistence after subclinical infection and lesion comparisons from patients suffering from *L*. *aethiopica* infections.

## Background

Leishmaniasis is a clinically and epidemiologically diverse disease caused by obligatory, intracellular, zoonotic hemo-flagellate protozoan parasites of the genus *Leishmania* (family Trypanosomatidae) [1-3]. It is transmitted to humans via bites of sandflies and is prevalent in 98 countries in the world [4]. Infection by the *Leishmania* parasite can cause either cutaneous leishmaniasis (CL) or systemic/visceral leishmaniasis (VL) [5]. CL is characterized by cutaneous lesions which develop at the site of the insect bite. Lesions can vary in severity, clinical manifestation, as well as recovery time, and in a proportion of patients, lesions can become chronic, leading to disfiguring mucosal leishmaniasis. CL can have a significant social impact as it may lead to severe stigmatization of affected individuals when lesions or scars occur on the face and exposed extremities [6]. CL is the most widely distributed form of leishmaniasis, with about one-third of the cases occurring in the Americas, the Mediterranean basin and Asia. Ten countries with the highest incidence rates are Afghanistan, Algeria, Colombia, Brazil, Iran, Syria, Ethiopia, North Sudan, Costa Rica and Peru, which together account for 70 to 75% of global estimates for CL [4]. A recent report indicates that about 20,000 – 50,000 CL cases are diagnosed each year [7]. In Ethiopia, leishmaniasis is present in both rural and urban areas and *Leishmania aethiopica* is considered the predominant etiological agent of CL. It causes local cutaneous leishmaniasis (LCL), mucocutaneous leishmaniasis (MCL) and diffuse cutaneous leishmaniasis (DCL) [8,9]. The recent increase in the number of reported CL cases in Ethiopia [10] as well as its diverse clinical manifestations highlight the epidemiological significance of the disease.

Standard diagnostic procedures for CL include detection of the parasite in a skin smear or biopsy using microscopy, or demonstration of the parasite in culture. However, even when these assays are combined, they are not sensitive enough to confirm all cases of CL. Serology is also an insufficient diagnostic tool for CL as systemic antibody responses are absent [11]. Furthermore, these techniques are unable to distinguish between different *Leishmania* species/strains that cause CL. Molecular techniques that detect parasite specific DNA or RNA offer definite advantages in sensitivity and speed of detection [12]. Such fast and accurate methods in the identification of disease causing parasites will further facilitate the delivery of appropriate treatment. These advantages make molecular methods a viable and attractive diagnostic strategy.

Recently, analysis of length polymorphisms of microsatellite-containing regions have become an important tool for population and genetic studies of different species [13]. Microsatellites are tandemly repeated stretches of short nucleotide motifs of 1 to 6 base pairs ubiquitously distributed in the genomes of eukaryotic organisms. They mutate at rates five to six orders of magnitude higher than that of the bulk of DNA. Microsatellite loci present high variability mainly due to allelic repeat length variations. The length variation of individual loci can easily be screened after amplification with primers that anneal specifically to their flanking regions [14]. *Leishmania* are relatively rich in microsatellites [15]. Multilocus microsatellite typing (MLMT) has been shown to be one of the best methods for distinction of *Leishmania* strains [16]. Previous population genetic studies performed by other researchers using MLMT revealed geographical and hierarchic population structures in *Leishmania major*, *Leishmania tropica* and the *Leishmania donovani* complex [16]. Microsatellite markers designed for species of *Leishmania* include 13 for *L*. *major*[17,18] 16 for *L*. *tropica*[19,20] and 20 for *L*. *donovani*[17]. Two independent microsatellite loci described by Rossi *et al*. [15] and three genomic fragments containing several different microsatellite tracts were found to be polymorphic in *Leishmania infantum*[21]. However, studies identifying microsatellite markers are not available for *L*. *aethiopica*. In this current study, we describe the characterization of molecular markers for *L*. *aethiopica* isolated from human CL patients.

## Methods

### *L*. *aethiopica* isolates

*L*. *aethiopica* parasites used for this study were obtained from the Department of Immunology, Microbiology and Parasitology, School of Medicine, College of Health Sciences, Addis Ababa University (AAU), Ethiopia. Samples of *L*. *aethiopica* parasites and or DNA were transferred to, and maintained at the Ohio State University following institutional guidelines. *L*. *aethiopica* isolates E, B, D, G and M were obtained from CL patients in Ethiopia and were also used in this study. *L*. *aethiopica* strain MHOM/ET/1972/L102 was used as a reference strain.

### DNA extraction

*L*. *aethiopica* parasites were maintained and cultured in Schneider's *Drosophila* medium supplemented with 20% fetal calf serum, 1% HEPES (4-(2-hydroxyethyl)-1-piperazineethanesulfonic acid), 10 U penicillin/ml, 100 μg streptomycin/ml and 0.05 mM 2-mercaptoethanol. Promastigotes were harvested by centrifugation and washed twice in PBS. DNA was extracted by the method described previously [22], resuspended in Tris-EDTA buffer (pH 7.4), and stored at 4°C.

### *L*. *aethiopica* species identification: PCR of ITS region and sequencing

The internal transcribed spacer in the ribosomal region was amplified with primers ITSFW (5′-ACACTCAGGTCTGTAAAC) and ITSRV (5′-CTGGATCATTTTCCGATG) as described previously [23]. In brief, a total of 10 ng template DNA in 2 μl was added to the reaction mixture containing 20 pmol of each primer in 5 μl, 12.5 μl Taq polymerase (Perkin-Elmer-Cetus, Norwalk, CT, USA) and 5.5 μl distilled water. PCR cycling conditions were as follows: initial denaturation of 95°C for 5 min, 35 cycles of 95°C for 20 s, 50°C for 30 s, 72°C for 1 min followed by a final extension of 72°C for 6 min. PCR products containing the amplified ITS region were separated in a 1% agarose gel with 1×TAE buffer and visualized under a UV transilluminator. Desired bands of expected sizes were excised and extracted using a Qiagen gel extraction kit (Valencia, CA, USA) according to the manufacturers’ instructions. Samples were submitted to the Plant-Microbe Genomics Facility at the OSU for sequencing. Sequence alignments were performed using the NIH based Basic Local Alignment Search Tool (BLAST) for identification of *Leishmania* species.

### Microsatellite library enrichment

We constructed an enriched genomic library based on the protocols developed by Bloor *et al*., [24]. Ten micrograms of genomic DNA extracted from *L*. *aethiopica* was digested with *HaeIII*. The fragments were ligated to specific adaptors consisting of “Oligo A” (5′-GGC CAG AGA CCC CAA GCT TCG-3′) and “Oligo B” (5′ PO_4_-GAT CCG AAG CTT GGG GTC TCT GGC C-3′) [25]. Fragments between 400 and 800 bp were excised from the agarose gel, extracted, and concentrated using YM-50 spin columns. The fractionated DNA was denatured and hybridized at a temperature of 55°C to (GT)_10_ 3′-biotinylated oligonucleotides bound to M-280 streptavidin-coated magnetic beads. After incubation at 72°C for 2 h, unbound DNA and excess oligonucleotides were removed following differential stringency washes. PCR amplification of the immobilized fragments was conducted by using a suspension of 1 mg magnetic beads containing enriched DNA fragments as template and 30 pmol of “Oligo A” primer. The terminal elongation step was extended to 30 min. The amplified fragments were ligated to TOPO vector and transformed into competent *E*. *coli*, according to the manufacturer's instructions (Life Technologies, Grand Island, NY). Cells were plated out on LB agar plates and incubated for overnight at 37°C. For screening of the library, colony PCRs were conducted by using a T_A_ of 55°C, 10 pmol of primers oligo A and (GT)_10_. Microsatellite-containing fragments produced double bands in subsequent gel electrophoresis. Plasmid DNAs from positive clones were sequenced at the Plant-Microbe Genomics Facility at the Ohio State University, to confirm the presence of microsatellites.

### Design of microsatellite markers

Using Primer3 software [26], PCR primers between 18 bp and 22 bp in length were designed to amplify sequences flanking the detected microsatellites. Primers were chosen from sequences 1 to 25 nucleotides upstream and downstream of the microsatellite repeat. A BLAST search was conducted for all markers to find corresponding sequences within the *Leishmania* genome to determine the chromosomes on which the amplified regions were localized.

### Analysis of microsatellite variation

Amplification reactions were performed using 40 ng *Leishmania* genomic DNA as template and 10 pmol of each primer. PCR products were screened for length variations by agarose gel electrophoresis in 4% MetaPhor agarose gels (Lonza Rockland, ME, USA), prepared according to the manufacturer's instructions.

### Ethical considerations

This study was reviewed and approved by the Institutional Review Board (IRB) of the School of Public Health, AAU. A Material Transfer Agreement (MTA) was signed between AAU and OSU.

## Results

### Identification of *L*. *aethiopica* from clinical isolates

**Figure 1:**
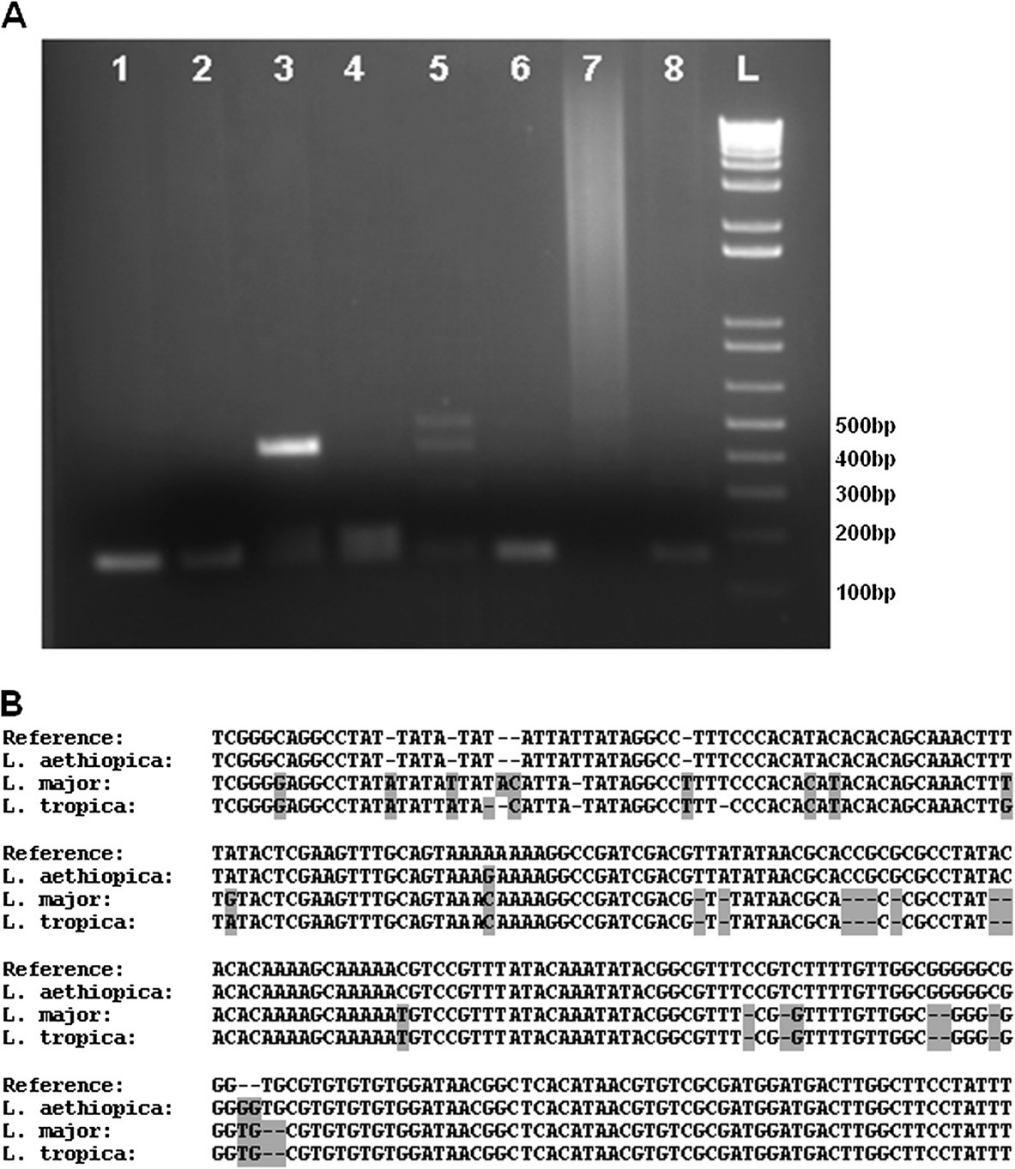
**Identification of *L. aethiopica *from clinical isolates. A**) PCR amplification of *Leishmania* genomic DNA from different species using previously developed primers MATRAE2 and Ae2.1 subjected to electrophoresis on a 1% agarose gel. Lanes 1 and 2 are PCR products from 2 different *L*. *aethiopica *isolates used in the study. Lanes 3, 4, 5, 6, 7 and 8 are PCR products amplified from genomic DNA of *L*. *donovani*, *L*. *major*, *L*. *mexicana*, *L*. *tropica*, *L*. *infantum and* mouse, respectively. L is 1kb DNA ladder. **B**) Basic Local Alignment search of sequences derived from PCR amplification of the ITS1 region of *L aethiopica*, *L major* and *L*. *tropica*, using L5.8S/LISTR primers. Genomic DNA from *L*. *aethiopica* MHOM/ET/1972/L102 was used as a reference strain.

Earlier work identified primers for identification of *L*. *aethiopica* from clinical isolates [25]. We were unable to differentiate *L*. *aethiopica* from other species of *Leishmania* (*L*. *tropica*, *L*. *major*, *L*. *mexicana*, *L*. *donovani* and *L*. *infantum*) with the use of these previously validated *L*. *aethiopica* specific primers (Figure 1A). On the other hand, although PCR analysis of the ITS1 region was unable to yield distinguishable bands using the *Leishmania* species used, sequencing of the PCR amplified ITS1 region and subsequent BLAST searches on genome databases identified *L*. *aethiopica* isolates. ITS1 sequences from *L*. *aethiopica* isolates used in this study were 99% homologous to *L*. *aethiopica* strains and shared 90% homology to *L*. *tropica*, and 83% to *L*. *major* sequences in genome databases (Figure 1B). We therefore utilized ITS1 gene sequencing for identification of all *L*. *aethiopica* isolates used in this study.

### Frequency and distribution of GT rich microsatellites

**Figure 2:**
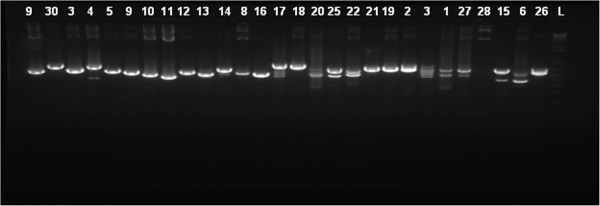
**Frequency and distribution of GT rich microsatellites. **PCR screening of select microsatellite containing *E*. *coli *clones using Oligo A and GT_10_ primers and transformed *E*. *coli *plasmid DNA as template. PCR products were electrophoresed on a 2% agarose gel. Clones that contained a microsatellite demonstrated a double band PCR product.

Previous methods developed for analysis of strain variation within *L*. *aethiopica* have not been very successful. We therefore examined whether the *L*. *aethiopica* genome was rich in microsatellites and determined the potential for utilizing microsatellites as a basis for discriminating between isolates of *L*. *aethiopica*. Other species of *Leishmania* have been shown to be rich in microsatellites and GT rich microsatellite markers have been identified for *L*. *major*[17], *L*. *tropica*[19] and *L*. *donovani*[19]. We therefore analyzed the frequency and distribution of GT rich microsatellites in *L*. *aethiopica* isolates. Following microsatellite enrichment, a total of 92 clones presumably containing GT rich microsatellite structures were obtained from the *L*. *aethiopica* (GT)_10_ enriched genomic library. Colony PCR of enriched clones using oligo A primers revealed a significant number of regions which were positive for GT containing sequences as shown by the presence of double bands after separation of PCR products on a 2% agarose gel (Figure 2). Subsequent DNA sequencing confirmed the presence of GT containing sequences in about 22 of these regions. While some of these sequences were homologous to other *Leishmania* species and could be mapped to specific chromosomes based on sequence alignment searches, other sequences seemed to be unique to *L*. *aethiopica* (Table 1).

**Table 1:** **Microsatellite markers of *L. aethiopica *developed and tested in this study**

**CO**	**REPEAT ARRAY**	**FORWARD PRIMER SEQUENCE (5′-3′)**	**REVERSE PRIMER SEQUENCE (5′-3′)**	**CHROMOSOME LOCATION**	**POLYMORPHISM**
1	(CA)10	TGAACAGTACGTCCAGGAGGA	GTGGTGGAAGAGGGAGAGAG	NA	P
3	(GT)20	TCTTGCTTACGCGTGGACTA	ACGAGGCAGGAGGGAGAG	Lmj11	NA
4	(CA)12	TGCGCCAGTTTCTATGACTG	TGTGTTTGTGTGCGTGGATA	Lmx32, Ld33	P
5	(GT)10	TTATGATGTAGGCAGGGTAGAGT	GTATTGGGCAAAGGGACCTT	Lmj31, Ld31	NA
6	(CA)29	TGCTCATGGTGGATGTGC	GTCGTGTGGTCGTGTATGTG	Lmj31	P
7	(CA)12	TTCGTCGCTCTTGTCTCTTG	TAGTCCACGCGTAAGCAAGA	Lmj24	NA
8	(CA)15	TCCCTCTCTTCCTCTGTTCC	CAGATGCGCAAGAATCAAAA	Lmj, Ld15	NA
11	(CA)16	CTCCCTCTCTTCCTCTGTTCC	TCTCTCGGTGAGCCAGATG	Lmj, Ld15	NA
13	(GT)16	GGTCGTTGTGACGCCAGT	ACTGCGTTCTGTGTGTCTCCT	Lmj31	NA
15	(GT)10	CCACCACCACACTTTTTCGT	GATACCGGCTGATGATGAGG	NA	NA
16	(GT)13	TCTTGCCTCTTTAATCTGTGTCC	GTTGTGGTGGTTGATCCGTT	Lmj34	NA
17	(CA)28	CTCACACATGGATGCAAAGC	CGTGTGTGTGTGCCATACAA	NA	NA
18	(GT)21	GGAGGGGGAGGAGTCTAAAA	TGTACTGCCGAGATTGAACG	Lmj34, Li31,34, Ld8, 34	NA
19	(GT)50, 30	TCTTGCTTACGCGTGGACTA	TAGAGTTGATTGCACGTGGC	Lmj12	NA
20	(GT)11	TACTTCGCGCACCGTCTCT	TTGTCGGTCGTGACTACTGC	Lmj26	NA
21	(GT)20	GCTTACGCGTGGACTACCA	CTATCGAGGGTCGGCAAC	Lmj11,26	NA
22	(GT)16	GGCGCGATATCCACTTCTT	TGTAAGTGTGTGGGTGGGTG	Lmj18, Lin18	P
23	(GT)12	GTGGGTTTTTGAATTATGTCGTC	GTATTGGGCAAAGGGACCTT	Lmj31, Ld31, Lin31, Lmx30	NA
26	(GT)15	TCTTGCTTACGCGTGGACTA	TAGAGTTGATTGCACGTGGC	Lmj12, Ld12	NA
27	(GT)40	CGTACGTCTGTTGCCTCTGT	CACTACTCCGCAGAACCGTA	Ld26	P
28	(GT)13	CTCTTGCTTACGCGTGGACT	AGAGACAAGAGCGACGAAGC	Lmj24	NA
30	(GT)16	CCTTTGCCTTCTCCCTTTTT	GAGCTTGGCGTAATCATGGT	NA	NA

Note: Lmj: *Leishmania major*, Lmx: *L. mexicana*, Lin: *L. infantum*, Ld: *L. donovani. *NA: not available.

### Identification of strains of *L*. *aethiopica* based on microsatellite typing

To determine whether polymorphisms in the GT containing microsatellites exist within strains of *L*. *aethiopica*, we designed primers flanking each of the 22 identified microsatellite sequences (Table 1). Primers were developed to align closely to the CA/GT repeats to reduce bias due to additional insertion/deletion events in the flanking regions. Furthermore, fragment length analysis in a MetaPhor agarose gel was used to elucidate the resulting short PCR products.

**Figure 3:**
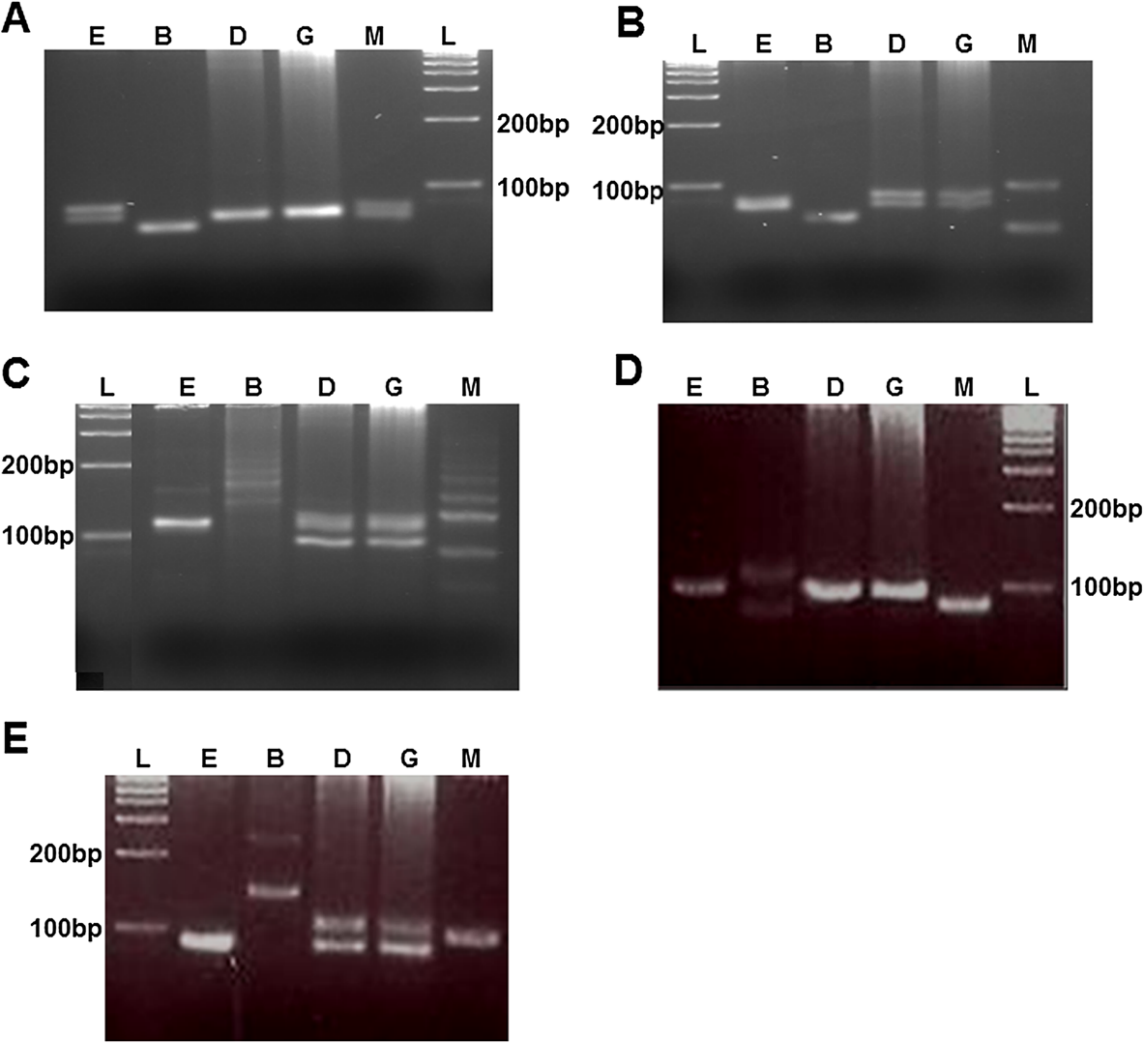
**Identification of strains of *L. aethiopica *based on microsatellite typing. **PCR analysis of genomic DNA extracted from various isolates of *L*. *aethiopica *using various markers designed from microsatellite regions identified in this study. PCR products were electrophoresed on a 4% Metaphor agarose gel and analyzed for fragment length variation. E, B, D, G and M represent different isolates of *L aethiopica *used in this study. **A**) Marker 1, **B**) Marker 4, **C**) Marker 6, **D**) Marker 22 **E**) Marker 27. L is 1 kb ladder.

All of the 22 microsatellite markers were tested with the different isolates of two previously identified strains of *L*. *aethiopica* in Ethiopia. The size of the amplified fragments (and thus the number of repeats) was compared to the size of fragments from cloned *L*. *aethiopica* strain MHOM/ET/1972/L102. Five of these markers demonstrated polymorphisms, as shown by variable PCR product sizes, within the species and strains of *L*. *aethiopica* (Figure 3). Microsatellite typing using these 5 markers further subdivided 2 previously identified strains of *L*. *aethiopica* into 4 groups. Two isolates (D and G) shared the same GT repeat profile while the other isolates were different based on their PCR product sizes.

### Identification of species specific primers for *L*. *aethiopica*

**Figure 4:**
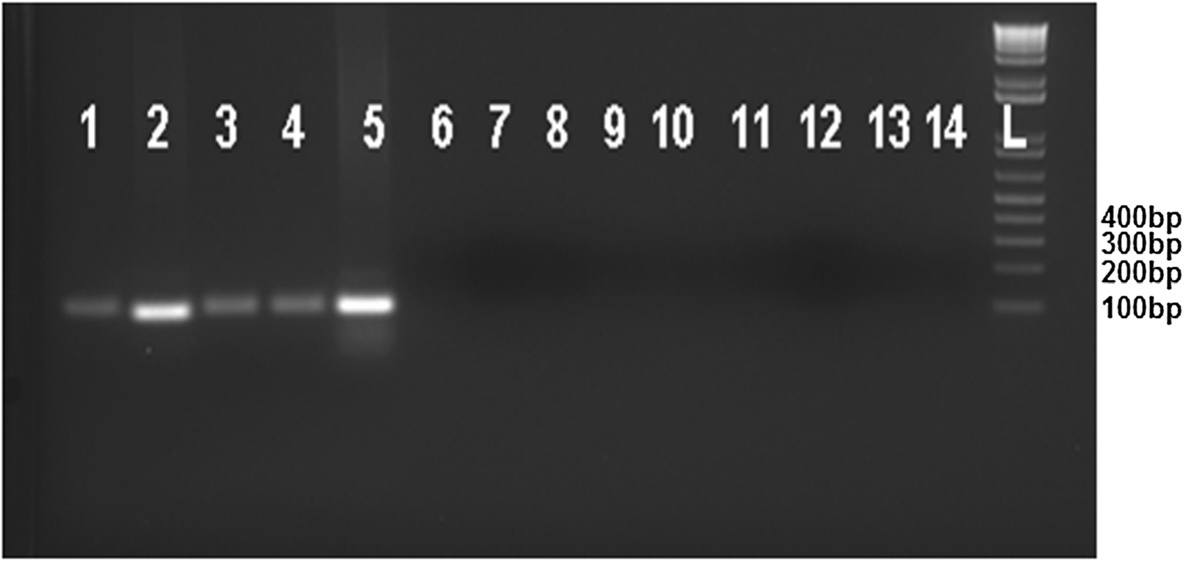
**Identification of species specific primers for *L. aethiopica. ***Multilocus microsatellite markers developed in the study analyzed for *Leishmania* species specificity. PCR analysis of genomic DNA extracted from different *Leishmania *species using primer 15 with an annealing temperature of 55.5°C. PCR products were electrophoresed on a 2% agarose gel and analyzed for the presence of a 100 bp band. Lanes: 1–5: different isolates of *L*. *aethopica*; 6: *L*. *donovani*; 7: *L*. *major*; 8: *L*. *mexicana*; 9–13:*L*. *tropica*; 14: *L infantum*. L represents 1 kb ladder.

Specie identification of clinical isolates from patients in Ethiopia has presented challenges. A number of PCR primers have been developed for identification of *L*. *aethiopica*[27], but we were unable to detect *L*. *aethiopica* specific bands using these primers (Figure 1A). We therefore tested PCR primers pairs which we had previously observed to be specific for *L*. *aethiopica* isolates. Our analysis revealed that PCR detection using primer 15 is specific for *L*. *aethiopica*. The specificity of this marker was evaluated using DNA extracted from different *Leishmania* species as well as from clinical isolates. DNA isolated from each species of *L*. *aethiopica* showed a 100 bp product after PCR amplification using primer 15, while DNA isolated from other species of *Leishmania* gave no PCR product under the same PCR conditions used (Figure 4). A PCR assay using this new primer could potentially be utilized as a tool for the diagnosis of CL due to *L*. *aethiopica* in patients.

## Discussion

In this study, we were able to identify hypervariable microsatellite loci and compile a set of markers usable for future epidemiological and population genetic studies for strains of *L*. *aethiopica*. Since this method is rapid and reproducible, we believe that it can be used for the reliable identification and characterization of *L*. *aethiopica* parasites. Of the 22 markers developed in this study, 5 polymorphic markers and one species specific marker were identified. MLMT has an advantage over other molecular techniques as results are reproducible and exchangeable between laboratories [16]. It has proved to be a powerful tool for population genetic investigations, as well as epidemiological investigations, of *Leishmania* species [21]. These short sequence repeats are highly polymorphic, codominant, and dispersed throughout the parasite genome. It has been shown that microsatellite loci of the family Trypanosomatidae are stable under laboratory conditions and can be detected directly in biological samples containing low amounts of parasitic DNA [21,28]. In addition, the results of microsatellite analysis are much easier to compare between laboratories and store in databases [29].

This present study using MLMT divided the available isolates of *L*. *aethiopica* into four clusters. Previous studies conducted using *L*. *aethiopica* isolated from the skin of patients indicated genetic variation within the species; Multilocus Enzyme Electrophoresis (MLEE) separated strains into two different genetic groups [23]. However, the techniques used suffer from poor reproducibility [29]. In addition polymorphic repeats are not conserved between different species of *Leishmania*[17]. Recently, analysis of length polymorphisms of microsatellite-containing regions has become an important tool for population and genetic studies for many different species [13,30]. Microsatellites are tandemly repeated stretches of short nucleotide motifs which mutate at rates of five to six orders of magnitude higher than that of the bulk of DNA and present high variability mainly due to allelic repeat length variation. The length variation of individual loci can easily be screened after amplification with primers that anneal specifically to their flanking regions [14]. These microsatellites are used for mapping genes in the genome because of their abundant distribution [31,32].

A number of researchers have developed microsatellite markers for different species of *Leishmania* including *L*. *major*[17,18], *L*. *tropica*[19,20] and *L*. *donovani*[17], as well as other organisms such as *Penicillium marneffei*[33], which are now available and used for MLMT. To our knowledge, this is the first report demonstrating the use of MLMT for the molecular characterization of *L*. *aethiopica*, a parasite which is highly prevalent in Ethiopia and the major cause of LCL, MCL and DCL, accounting for an annual incidence of 50,000 cases [4].

Feasibility of high-throughput MLMT requires the optimization of PCR product analysis. In this study we show that MetaPhor gel electrophoresis and sequencing both produced analogous and reproducible results. Sequencing was used to determine the number of repeats. This is indispensable for the analysis of large fragments containing more than one microsatellite. However, this method is expensive and sequences containing small tandem repeats may be difficult to process. We demonstrate in this study that using MetaPhor agarose gel electrophoresis to screen for polymorphisms produces sufficient resolution to distinguish between *L*. *aethiopica* strains and could identify short tandem repeats [29].

All of the *L*. *aethiopica* isolates tested showed exclusive multilocus microsatellite patterns using the five identified markers. Thus MLMT could potentially enable researchers to potentially track strains of this parasite, making this an effective epidemiological tool. Even with these promising results, however, more isolates will need to be processed to confirm the spatial clusters or subdivisions of *L*. *aethiopica* in Ethiopia. Studies conducted on *L*. *aethiopica* are few compared to other species of *Leishmania* possibly due to its prevalence restricted to the eastern part of Africa. This study provides tools that will enable further molecular epidemiological and population genetic research on CL caused by *L*. *aethiopica*.

Primers previously developed for *L*. *aethiopica* were unable to identify the different *Leishmania* species and isolates in our study. We therefore utilized the previously characterized ITS1 regions to identify and confirm the identity of the *Leishmania* species used in this study. Sequencing of PCR products generated using ITS1 specific primers and performing sequence alignments against the *Leishmania* genome database enabled us to identify these isolates. The species specific primers developed in our current study could provide a quicker, cost effective and highly useful tool for the typing/diagnosis of *L*. *aethiopica* on clinical samples. This would be useful for case detection, determination of appropriate therapeutic regimens as well as implementation of control measures. Further, since this method does not require a restriction enzyme digestion step as in restriction fragment length polymorphism (RFLP), it provides an added advantage in accelerating species identification.

## Conclusion

In conclusion, we demonstrate in this study, the successful development of markers for multilocus microsatellite typing of strains of *L*. *aethiopica*. We further successfully designed a species specific marker for *L*. *aethiopica*. The MLMT markers developed in this study have great potential for use in epidemiological and population genetic studies of strains of *L*. *aethiopica*. It will potentially facilitate investigation of the structure and dynamics of the corresponding natural foci. It will also help to answer specific clinical questions, such as the occurrence of local and diffuse lesions, strain correlates of parasite persistence after subclinical infection, lesion comparisons from patients suffering from *L*. *aethiopica* infections and the determination of endogenous and/or exogenous reinfection associated with immunosuppression.

## Competing interest

The authors declared that they have no competing interest.

## Authors’ contributions

NK, SO, ARS, AW, AH conceived the study. NK, SO, ARS designed the experiments. NK, SO, SV performed the experiments. NK, SO, ARS, AW, AH analyzed the data. NK, SO, ARS prepared the paper. All authors contributed to the writing of the manuscript and approved the submitted version of the manuscript.
